# TRiCit: A High-Throughput Approach to Detect *Trichomonas vaginalis* from ITS1 Amplicon Sequencing

**DOI:** 10.3390/ijms241411839

**Published:** 2023-07-24

**Authors:** Mykhaylo Usyk, Nicolas F. Schlecht, Shankar Viswanathan, Ana Gradissimo, Negin Valizadegan, Christopher C. Sollecito, Anne Nucci-Sack, Angela Diaz, Robert D. Burk

**Affiliations:** 1 Department of Pediatrics (Genetic Medicine), Albert Einstein College of Medicine, Bronx, NY 10461, USA; mykhaylo.usyk@einsteinmed.edu (M.U.); gradisa@mskcc.org (A.G.); valizad2@illinois.edu (N.V.);; 2 Department of Epidemiology and Population Health, NYU Grossman School of Medicine, New York, NY 10016, USA; 3 Department of Cancer Prevention & Control, Roswell Park Comprehensive Cancer Center, Buffalo, NY 14263, USA; nicolas.schlecht@roswellpark.org; 4 Department of Epidemiology and Population Health, Albert Einstein College of Medicine, Bronx, NY 10461, USA; 5 Department of Pediatrics, Mount Sinai Adolescent Health Center, Icahn School of Medicine at Mount Sinai, Manhattan, NY 10029, USA; 6 Departments of Microbiology and Immunology, and Obstetrics and Gynecology and Women’s Health, Albert Einstein College of Medicine, Bronx, NY 10461, USA

**Keywords:** *Trichomonas vaginalis*, ITS, mycobiome, amplicon, *molBV*, molecular diagnosis

## Abstract

Trichomoniasis, caused by *Trichomonas vaginalis* (TV), is the most common non-viral sexually transmitted infection (STI) worldwide, affecting over 174 million people annually and is frequently associated with reproductive co-morbidities. However, its detection can be time-consuming, subjective, and expensive for large cohort studies. This case–control study, conducted at the Mount Sinai Adolescent Health Center in New York City, involved 36 women with prevalent TV infections and 36 controls. The objective was to examine Internal Transcribed Spacer region-1 (ITS1) amplicon-derived communities for the detection of prevalent TV infections with the same precision as clinical microscopy and the independent amplification of the TV-specific TVK3/7 gene. DNA was isolated from clinician-collected cervicovaginal samples and amplified using ITS1 primers in a research laboratory. Results were compared to microscopic wet-mount TV detection of concurrently collected cervicovaginal samples and confirmed against TV-specific TVK3/7 gene PCR. The area under the receiver operating characteristics curve (AUC) for diagnosing TV using ITS1 communities was 0.92. ITS1 amplicons displayed an intra-class correlation coefficient (ICC) of 0.96 (95% CI: 0.93–0.98) compared to TVK3/7 PCR fragment testing. TV cases showed an increased risk of bacterial vaginosis (BV) compared to the TV-negative controls (OR = 8.67, 95% CI: 2.24–48.54, *p*-value = 0.0011), with no significant differences regarding genital yeast or chlamydia infections. This study presents a bioinformatics approach to ITS1 amplicon next-generation sequencing that is capable of detecting prevalent TV infections. This approach enables high-throughput testing for TV in stored DNA from large-scale epidemiological studies.

## 1. Introduction

Trichomoniasis caused by infection with the eukaryotic protozoa *Trichomonas vaginalis* (TV) is currently the most common non-viral sexually transmitted infection, affecting an estimated 174 million people globally, with over half of these cases occurring in resource-limited regions [1,2]. TV infection is often related to reproductive sequelae, including co-infection with chlamydia [3], preterm birth [4], and miscarriage, and it has even been suggested to be involved in an increased risk of HIV transmission [5]. It is thus an infection of clinical and epidemiological relevance, but there is a limited capacity to detect it using cost-effective techniques, particularly in areas where clinical infrastructure is lacking and this condition is most prevalent.

In the clinical setting, TV is diagnosed using wet-mount microscopy, but this approach is not practical for large-scale studies. Molecular techniques offer an alternative with rRNA-based nucleic acid amplification test (NAAT) [6] or DNA polymerase chain reaction (PCR) assays being available for TV detection [7] via TV-specific gene targets (i.e., the TVK3/7 gene [8]). While this allows for high-throughput screening of a population, a major limitation of these approaches is that they can detect only TV infection, making them less efficient than approaches that can more broadly provide information on the microbiome composition.

In light of these considerations, we aimed to assess the ability of an Internal Transcribed Spacer 1 (ITS1) amplicon Next-Generation Sequencing (NGS) assay to identify Trichomonas vaginalis (TV) infections. TV is a eukaryotic organism, meaning that standard bacterial 16S ribosomal RNA (rRNA) gene amplification assays, commonly used in identifying bacterial species, are not applicable as TV lacks this specific gene. On the other hand, the ITS1 region, which is an area between the 18S and 5.8S rRNA genes in the eukaryotic ribosomal gene cluster, is present not only in TV but also in other common vaginal pathogens such as yeast [9]. Thus, an assay targeting the ITS1 region would be highly efficient in detecting TV infections as it would do so while simultaneously mapping the overall composition of the cervicovaginal mycobiome.

In this study, we present a bioinformatics approach that uses next-generation sequencing (NGS) data from the 48F and 217r primers previously designed to profile human fungi that comprise the mycobiome [9]. This approach detected the TV ITS1 fragment with the same precision as a separate next-generation sequencing assay targeting the TVK3/7 gene (the current gold standard for molecular TV detection). We present a protocol for using ITS1 to detect TV. The current study presents data that demonstrates that the use of ITS1 for TV detection is specific and cost-effective for large epidemiological cohorts.

## 2. Results

### 2.1. Patient Characteristics

The demographic characteristics of the 72 TV case–control participants are summarized in Table 1. The cases were enrolled with a median (IQR) age of 20.1 (3.3), which was comparable to controls that had a median (IQR) age of 20.3 (3.1). There were no differences between the patients regarding rates of genital warts or prevalent chlamydia infections. Bacterial vaginosis (BV) however, showed substantial differences, with the cases showing simultaneously higher rates of BV-positive and lower rates of BV-negative states as measured using the molecular BV (*molBV*) score derived using 16S [10] (OR = 8.67, 95% CI: 2.24–48.54, *p*-value = 0.0011). The BV-intermediate category was also significantly elevated in TV cases as compared to controls, OR = 6.12, 95% CI: 1.28–39.55, *p*-value = 0.023.

### 2.2. ITS1 and 16S Derived Communities in Trichomonas vaginalis Infection

**Figure 1:**
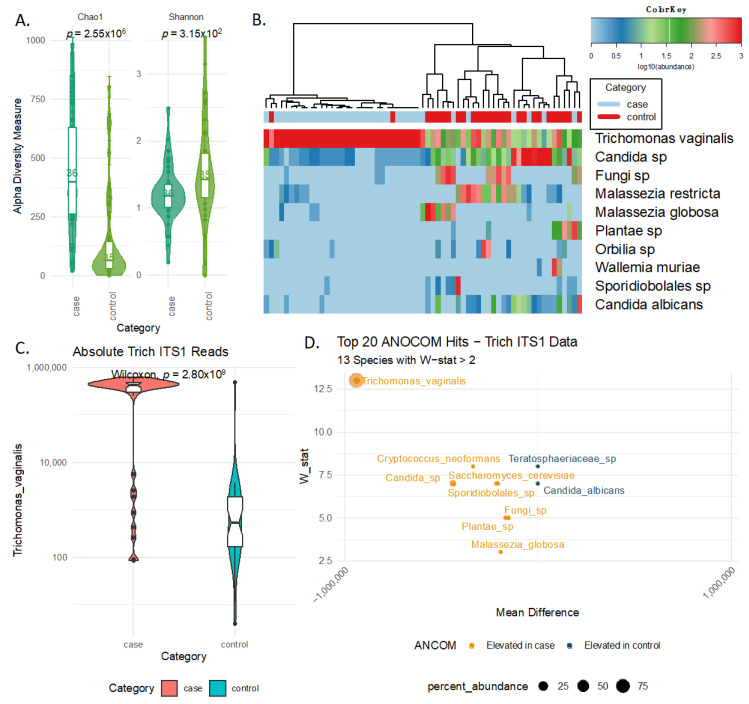
ITS1-Derived Eukaryotic Community is Fundamentally Altered in TV Cases. Panel (**A**) shows the ITS1 alpha diversity with respect to Chao1, which is a measure of richness (i.e., how many different species are detected) and Shannon diversity, which is a measure of evenness (i.e., how uniformly the sequencing reads are spread across the different sequence variants). *p*-values in the panel are based on *t*-test. Panel (**B**) shows a heatmap of the top 10 species detected using ITS1, with the top strip showing the TV case status (red for controls and blue for cases). The dendrogram above the heatmap is constructed using the Euclidian distances of the top 20 species and clustered using ward.D2 algorithm. Panel (**C**) compares the absolute resolve of ITS1 TV reads by clinical TV status with *p*-values being calculated using the *t*-test. Panel (**D**) shows the differential abundance analysis derived using ANCOM based on the TV case status.

Following the characterization of the vaginal mycobiome, we noticed major differences between TV cases and controls in all of the considered measures (Figure 1). In terms of the overall alpha diversity (Figure 1A), the cases had a significantly higher total amount of eukaryotic species detected using the ITS1 assay (Chao1 *p*-value < 0.001). After correction for diversity richness, differences were reversed due to the overabundance of TV in the case samples (Shannon *p*-value = 0.032). Figure 1B shows the top 10 species (based on total cohort read recovery) and illustrates that the cases have most of the sequencing reads matching *Trichomonas vaginalis* (line 1 of the heatmap). In fact, the detection of TV appears to be the primary driver of the cluster dendrogram presented on top of the heatmap that separates samples into those dominated by TV and those with a more balanced spread between the top species. Over three-quarters (n = 25/36) of cases fell into the TV-dominated clade, whereas 30/36 controls fell into the higher diversity clade (Chi-square *p*-value < 0.001). Considering just the ITS1 TV read recovery, cases had higher overall levels of *TV* with a median (IQR) recovery of 418,412 (178,824) reads/case and 530 (1697) reads/control (Figure 1C, *t*-test *p*-value < 0.001). Analysis of the compositions of microbiomes with bias correction (ANCOM), a microbiome-specific differential abundance analysis approach, also revealed *TV* as the top differential species detected using ITS1 (Figure 1D). ANCOM also indicated that cases had a higher proportion of several fungal species, including *Cryptococcus neoformans* and *Saccharomyces cerevisiae* (Figure 1D). Conversely, the controls showed elevated levels of an unidentified species of the family *Teratosphaeriaceae* and *Candida albicans.*

**Figure 2:**
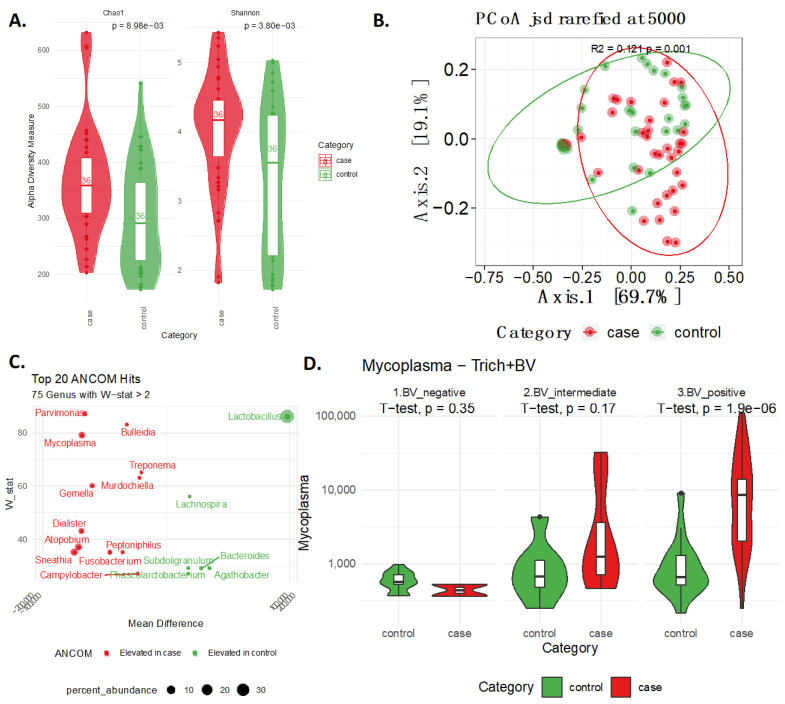
The CVM is Fundamentally Altered Based in TV Cases. Panel (**A**) shows the bacterial alpha diversity for Chao1 and Shannon indices, *p*-values were calculated using the *t*-test and are shown at the top. Panel (**B**) shows a principal coordinate analysis (PCoA) performed using Jensen–Shannon divergence with effect size and significance computed using PERMANOVA. Panel (**C**) shows bacterial differential abundance analysis performed using ANCOM with the top 20 differential genera plotted (based on W-stat). Panel (**D**) shows *Mycoplasma* abundance based on TV case status and BV strata from negative to positive with *p*-values calculated using the *t*-test shown at the top.

In terms of bacterial communities, TV cases showed a significant increase in alpha diversity in terms of both richness and evenness (Chao1 and Shannon *p*-values = 0.0090 and 0.0038, respectively, Figure 2A). Additionally, beta-diversity analysis using Jenson Shannon Divergence indicated consistent community differences between cases and controls in terms of bacterial genera (R^2^ = 0.121, *p*-value < 0.001, Figure 2B). Differential abundance further confirmed this via the identification of a total of 75 differential bacterial genera between case–control groups, including *Lactobacillus* (higher in controls) and *Parvimonas*, *Bulleidia*, *Mycoplasma,* and *Treponema* (higher in cases) (see Figure 2C). Of note, *Mycoplasma* is particularly interesting given the known symbiotic relationship between TV and *Mycoplasma* [11]. To explore whether this symbiotic association was related to BV being overrepresented in *TV* cases (see Table 1), we stratified samples by *molBV* categorical (Nugent score like) states (i.e., *molBV*-negative, *molBV*-intermediate, and *molBV*-positive). *Mycoplasma* was significantly elevated in the *TV* cases as compared to controls, even when the controls were *molBV*-positive (log2-FC = 3.60, *p*-value < 0.001, Figure 2D). Thus, the presence of *Mycoplasma* and TV was not simply related to the co-occurrence of TV and BV.

### 2.3. Defining Molecular ITS1 Thresholds of Trichomonas vaginalis Detection

**Figure 3:**
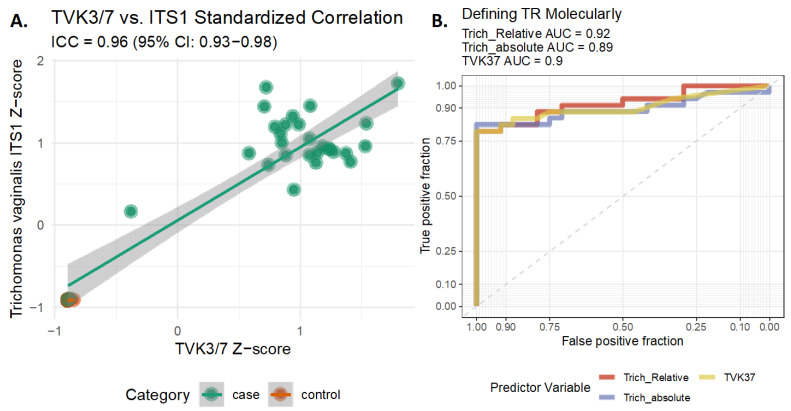
ITS1 and TVK3/7 TV PCR Marker Gene Comparison. Panel (**A**) shows the scatter plot of the normalized NGS recovery of ITS1 TV reads compared to the TVK3/7 recovery. Consistency was calculated using two-way mixed effect intra-class correlation coefficient (ICC) on z-score standardized ITS1 TV NGS recovery and TVK3/7 recovery. Panel (**B**) shows the AUC analysis for the relative and absolute abundance of TV measured using ITS1, as well as the same analysis performed using the TVK3/7 NGS recovered reads. AUC for each metric is shown above the plot.

To confirm the presence of TV in clinical samples, we used a TV-specific TVK3/7 gene amplification assay [8]. There was a remarkably higher recovery of TVK3/7 reads in the clinical TV cases with a median (IQR) recovery of 39,014 (12,009) reads/sample vs. 2 (2.5) reads/sample in controls (*p*-value < 0.001). Comparing the TVK3/7 read recovery to that of ITS1 TV-reads revealed a similar pattern (Pearson correlation = 0.96, *p*-value < 0.001, Figure 3A).

After the additional ITS1 validation using the TVK3/7 PCR assay, we defined the ITS1 thresholds of TV detection using a molecular/bioinformatic approach we designate as TRiCiT. In addition to comparing the AUCs for the prediction of absolute ITS1 TV and TVK3/7 recovery, we also considered the relative abundance of ITS1 TV reads. The analysis revealed that all three measures (see Figure 3B) provided strong predictive power for clinical TV identification, with the relative ITS1 TV showing the best performance with an AUC of 0.92 (Figure 3B). Optimizing the sensitivity and specificity across the AUC curve identified the optimal cut-point of the relative ITS1 TV reads to be 95.2%. For full details on using ITS1 to obtain a TV diagnosis, see methods Section 4.7.

## 3. Discussion

This study presents a high-throughput molecular assay and bioinformatics pipeline, TRiCiT, which leverages ITS1 amplicon-derived analyses to detect prevalent TV infection with the same precision as clinical microscopy and of the TVK3/7 gene PCR assay (AUC > 0.9). This approach offers significant potential for large cohort studies, particularly where cervicovaginal DNA is available. The ITS1-based assay and TV pipeline described in this report represents a significant advancement, as it simultaneously detects TV and distinguishes amongst a broad spectrum of fungi and yeast that are part of the mycobiome [12]. Hence, by targeting the ITS1 region, this assay is capable of identifying a vast array of eukaryotic organisms and potential pathogens, including those causing diseases that often have overlapping symptoms. The advantage here is twofold. First, it provides the potential for a rapid research diagnosis, which is essential for understanding the epidemiology of TV. Second, it reduces the need for multiple organism-specific tests, thus making the process more efficient and cost-effective.

Our results were validated using an independent NGS assay targeting the TV-specific TVK3/7 gene. Specifically, we observed a strong correlation between TRiCiT’s ITS1 amplicon-derived TV content and the TV-specific TVK3/7 PCR fragment analyses (molecular gold standard) that achieved an ICC of 0.96 (95% 0.93–0.98), indicating highly concordant results despite the two tests targeting different genomic loci within TV. The high AUC (0.92) based on clinical diagnosis for detecting TV using ITS1 and the TRiCit pipeline further supports the assay’s robustness.

Additionally, we performed an analysis of molecular bacterial vaginosis (BV) to evaluate the context of the cervicovaginal ecosystem [13]. We observed that TV cases had dramatically higher rates of BV compared to the controls (OR = 8.67, 95% CI: 2.24–48.54, *p*-value = 0.0011). This finding aligns with prior research [14,15,16], suggesting that TV infections frequently co-occur with other STIs and can lead to reproductive health issues [17]. Moreover, our study revealed a strong correlation between TV infection and elevated levels of *Mycoplasma*, a symbiotic bacteria that infects TV [11]. The identification of these known associations (i.e., BV and *Mycoplasma*) further supports the validity of detecting TV using the TRiCiT pipeline.

Nevertheless, the study does have limitations. The sample size is somewhat limited and based on a specific population. Whether the TRiCiT pipeline extends to all populations will require further analyses, although we have unpublished preliminary data that the method is valid across other populations from different continents. In addition, the association of TV and BV could be related to subsample selection; however, other studies have also detected BV in women with TV, as discussed above. Finally, the proposed detection approach is limited to the detection of clinical TV infection but is not adequate for the stratification of patients on the basis of resistance to antiprotozoal drugs.

In summary, the TRiCiT assay presents a relatively simple approach for detecting prevalent TV infection consistent with clinical testing via wet-mount and TV-specific TVK3/7 gene amplification. Its potential for efficient evaluation of TV’s impact on routine vaginal mycobiome measurements, particularly in large epidemiological studies, makes it a valuable tool for future research to address the burden of trichomoniasis on the reproductive health of women.

## 4. Methods

### 4.1. Study Cohort

Participants were selected from an HPV vaccine longitudinal cohort study [18] of sexually active adolescent female patients receiving gynecological care at Mount Sinai Adolescent Health Center (MSAHC) in New York City. Overall population characteristics, eligibility criteria, and study design of the larger cohort have been previously described [18]. Briefly, study participants were enrolled between the ages of 13 and 21 and received a gynecological examination at each study visit, approximately every six months. Routine screening for gonorrhea and chlamydia was performed about every six months and as clinically indicated for TV (e.g., symptoms of vaginal discharge [19]) using wet mounts. Research questionnaires were administered at every study visit, which included questions on sexual behaviors, sexual partners, history of STIs, condom use, drug and alcohol use, and other related factors. Written informed consent was obtained from all participants prior to enrollment. The Institutional Review Board at Mount Sinai School of Medicine approved the study. Controls were selected from a random individual from the same sampling visit that did not have TV.

### 4.2. Microbiome Sample Collection and DNA Extraction

Banked DNA samples for analysis of the CVM were obtained from specimens collected at the cohort study visits using a cervical Cytobrush^®^ placed in a PreservCyt transport medium (ThinPrep^®^; Hologic, Marlborough, MA, USA) following the same procedure as for Pap smears. Cervical samples were stored immediately at −20 °C until transport to the research lab at the Albert Einstein College of Medicine. The samples were transferred to a 15 mL tube in the lab and gently centrifuged at 1500 RPM for 5 min. After removing the supernatant via decanting, the pellets were rinsed in 3 mL of TE (10 mM Tris, 1.0 mM EDTA). This solution was then vortexed and centrifuged at 1500 RPM for 5 min, and the supernatant was removed via decanting. The remaining pellet and leftover solution (~150 µL) were used for DNA isolation via column processing with the QIAamp Mini spin column (Qiagen, Santa Clarita, CA, USA) following the manufacturer’s protocol. The purified DNA was eluted in 150 µL of elution buffer (10 mM Tris/0.5 mM EDTA, pH 9) and used initially for HPV DNA analyses and then stored at −20 °C in a non-frost free freezer.

### 4.3. PCR Amplification

PCR for bacterial communities was performed using forward (515F) GTGYCAGCMGCCGCGGTA and reverse (806R) GGACTACHVGGGTWTCTAAT primers that amplify the V4 hypervariable region of the prokaryotic 16S rRNA gene [20,21]. All primers contained unique Golay barcodes to allow for dual indexing of each sample. PCRs were conducted in a 25 µL reaction with 2 µL input of template DNA, 16.75 µL of ddH20, 2.5 µL of Platinum 10× PCR buffer (Invitrogen, Waltham, MA, USA), 0.75 µL of MgCl_2_ (50 mM, Invitrogen), 0.5 µL of dNTP mix (10 mM each, Roche, Basel, Switzerland), 0.25 µL AmpliTaq Gold, polymerase (5 U/µL, Applied Biosystems, Carlsbad, CA, USA), 0.25 µL of Platinum Taq DNA Polymerase (10 U/µL, Invitrogen), and 1 µL (5 µM) of each primer (IDT, Coralville, IA, USA). Thermocycling conditions included an initial denaturation at 95 °C for 5 min, followed by 15 cycles of 95 °C for 1 min, 55 °C for 1 min, 72 °C for 1 min, followed by 15 cycles of 95 °C for 1 min, 60 °C for 1 min, 72 °C for 1 min, and a final extension at 72 °C for 10 min.

PCR for fungal communities was performed using barcoded forward (48F) ACACACCGCCCGTCGCTACT and reverse (217R) TTTCGCTGCGTTCTTCATCG primers that amplify the ITS1 region of the prokaryotic ribosomal gene cluster [9,22]. PCRs were conducted in a 25 µL reaction with 10 µL input of template DNA, 8.75 µL of ddH20, 2.5 µL of Platinum 10× PCR buffer (Invitrogen), 0.75 µL of MgCl_2_ (50 mM, Invitrogen), 0.5 µL of dNTP mix (10 mM each, Roche), 0.25 µL AmpliTaq Gold polymerase (5 U/µL, Applied Biosystems), 0.25 µL of Platinum Taq DNA Polymerase (10 U/µL, Invitrogen), and 1 µL (5 µM) of each primer (IDT, Coralville, IA, USA). Thermocycling conditions included an initial denaturation at 95 °C for 5 min, followed by 35 cycles of 95 °C for 30 s, 55 °C for 30 s, 72 °C for 2 min, followed by a final extension at 72 °C for 10 min. All PCRs were conducted in a GeneAmp PCR System 9700 (Applied Biosystems), and PCR products were verified via gel electrophoresis.

### 4.4. NGS Library Preparation and Sequencing

PCR products for each sample were pooled via PCR assay (16S and ITS1) in approximately equal concentrations, and 100 µL of the pooled products were loaded into a 3% agarose gel and run at 80 V for 3 h to separate the DNA fragments. The DNA fragment for each assay was excised and purified with a QIAquick Gel Extraction Kit (Qiagen) and quantified using a Qubit High-Sensitivity dsDNA assay (Invitrogen). NGS library preparation was conducted on the purified pooled PCR products from each assay with a KAPA LTP Library Preparation Kit (KAPA Biosystems, Wilmington, MA, USA) according to the manufacturer’s protocol. The library amplicons were validated on a 2100 Bioanalyzer (Agilent Technologies, Santa Clara, CA, USA), and sequenced at PE150 bp carried out on an Illumina HiSeq4000 at Genewiz/Azenta (South Plainfield, NJ, USA).

### 4.5. Bioinformatic Analysis

Sequence reads (for 16S analysis) were clustered into amplicon sequence variants (ASVs) using DADA2, and taxonomy was assigned using a custom cervicovaginal microbiome-specific database [10] employing a Naive Bayesian classifier. We assessed the presence of bacterial vaginosis (BV) using a previously validated molecular score from the 16SV4 reads (*molBV*) that provide a Nugent-like score on a scale from 0 to 10 [10]. Cervicovaginal samples with *molBV* scores of 0–3 were classified as *molBV* BV-negative, those with >3–≤7 were classified as *molBV* BV-intermediate, and those with >7–10 were classified as *molBV* BV-positive. Cervicovaginal microbiome community state types (CSTs) were generated using VALENCIA [23]. 

Negative controls (n = 17) and positive controls (n = 17) were used to control for contamination and downstream amplification, sequencing, and bioinformatics, respectively. In terms of the negative controls, 16S rRNA sequencing showed an average (SD) read recovery of 2803 (1000) reads vs. true samples 36,384 (13,512) (*t*-test *p*-value < 0.001). For ITS1 sequencing, the recovery was 1741 (4563) reads in the negative controls and 7774 (20,879) in true samples (*t*-test *p* < 0.001). The negative control was water. The positive control was purchased from ZymoBIOMICS Microbial DNA Standard (Zymo Research Corp., Irvine, CA, USA) and, after amplification and sequencing, showed the expected composition, including the bacteria—*Pseudomonas aeruginosa*, *Escherichia coli*, *Salmonella enterica*, *Lactobacillus fermentum*, *Enterococcus faecalis*, *Staphylococcus aureus*, *Listeria monocytogenes* and *Bacillus subtilis*, and fungi—*Saccharomyces cerevisiae* and *Cryptococcus neoformans*.

### 4.6. Statistical Analyses

The difference in the distribution of continuous variables was analyzed using the *t*-test. Alpha diversity and Jenson–Shannon divergence were calculated using the phyloseq package [24]. Beta diversity significance was assessed using PERMANOVA using the vegan package [25]. Consistency was assessed using two-way mixed effects intra-class correlation coefficient (ICC) between the standardized TV ITS1 recovered reads and the TVK3/7 amplicons due to the variability in scale units. The area under the curve analysis was performed using the pROC and caret packages [26,27]. The optimal cutpoint for identifying TV cases using ITS1 sequence data (i.e., TRiCiT) was performed by maximizing the sensitivity and specificity. A multivariable exact logistic regression model was fitted to assess the association between TV and molBV categories.

### 4.7. TRiCiT Diagnosis

**Figure 4:**
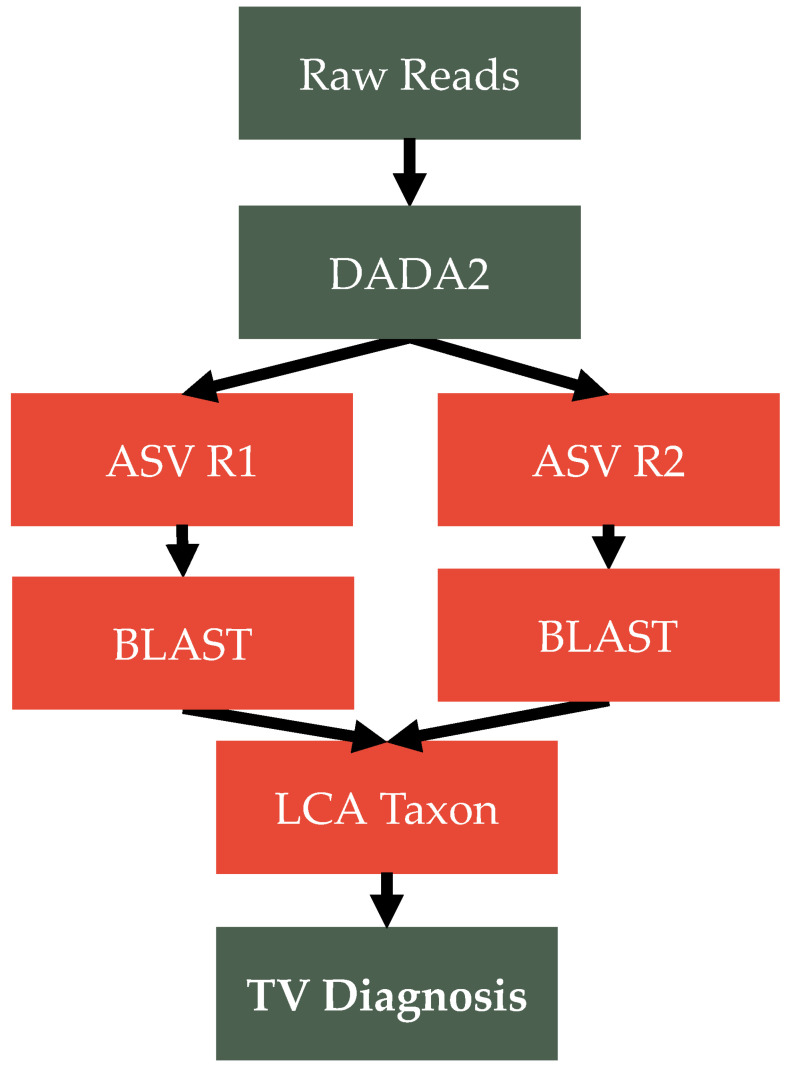
TRiCiT Pipeline Overview. Figure shows the steps involved in diagnosing TV using the ITS1 assay. First, the raw reads are concatenated and processed using DADA2 using default settings. Reads are then split into R1 and R2 in order to perform taxonomic classification using BLAST and the UNITE database V9 expanded to include the ITS1 sequence of Trichomonas vaginalis (NCBI Accession: L29561.1). Taxonomy is then consolidated between R1 and R2 by taking identification that either reaches a percent identity match >90% or the lowest common ancestor between the two segments of the ASV if neither/both achieve said similarity threshold. The ITS1 reads of TV are then extracted, and a TV diagnosis is assigned for any sample that achieves a relative TV abundance of 95.2% or more and has a minimum recovery of at least 100 reads or until a rarefaction threshold is reached within a given ITS1 community (to ensure that samples have reliable measurement).

Obtaining a TV diagnosis using ITS1 sequencing amplicons requires two primary steps (Figure 4):

(1) Processing ITS1 reads using standard ASV approaches and (2) using quantified Trichomonas ITS1 levels to identify TV cases.

For step 1, ITS1 amplicons are processed using DADA2 [28] with default settings except for enabling the concatenation options. Reads are concatenated because ITS1, unlike 16S regions, is highly variable in size (can range as much as 300–700+ bp [9]), and so it is impossible to predict whether DADA2 will be able to correctly merge all reads without knowing the full ITS1 composition of the sample prior to running it. Following DADA ASV generation, the reads are split back to R1 and R2 segments and classified taxonomically using BLAST [29] and the UNITE V9 ITS1 database [30] that has been expanded to include the TV ITS1 sequence (NCBI Accession: L29561.1). The classification of each ASV segment is then “stitched” back together by taking the result that achieves a 95% similarity threshold or the lowest common ancestor (LCA) of R1/R2 if neither/both achieve this similarity level.

For step 2, the ITS1 of TV is extracted, and the identified relative cut-off is used based on the AUC analysis outlined in the result Section 2. Specifically, a sample is defined as being TV positive if it has a relative proportion of TV 95.2%. Additionally, a sample must exceed a minimum sequencing threshold of 100 reads (or the rarefaction threshold is for the tested cohort). The latter criterion is added to ensure that a TV diagnosis is not assigned on the basis of shallow sequencing depth samples (i.e., without a coverage threshold, a sample may be assigned a positive diagnosis on the basis of a single read, which would represent 100% TV detection).

## Figures and Tables

**Table 1:** Distribution of Participant Characteristics by Case-Control Status.

Variable	Case	Control	OR	*p*-Value
N	36	36		
Median (IQR)	20.1 (3.3)	20.3 (3.1)	-	0.65
BV diagnosis (*molBV*)				
BV negative (ref.)	2 (5.6%)	13 (36.1%)	-	-
BV intermediate	8 (22.2%)	7 (19.4%)	6.12 (1.28–39.55)	0.023
BV positive	26 (72.2%)	16 (44.4%)	8.67 (2.24–48.54)	0.0011
Genital warts				
Negative (ref.)	33 (94.3%)	35 (100%)	5.29 (0.41–741.37)	0.22
Positive	2 (5.7%)	0 (0%)	-	-
Missing (n = 2)				
Chlamydia				
Negative (ref.)	34 (94.4%)	35 (97.2%)	1.71 (0.22–19.44)	0.61
Positive	2 (5.6%)	1 (2.8%)	-	-
Yeast				
Negative (ref.)	29 (87.9%)	2 (66.7%)	-	-
Positive	4 (6.1%)	4 (33.3%)	0.28 (0.043–1.95)	0.18
Missing (n = 33)				

Table shows the univariate unadjusted associations between participant characteristics and clinical *Trichomonas vaginalis* detection by wet mount preparation. Odds ratios (ORs) and 95% confidence intervals and *p*-values were estimated using Exact logistic regression.

## Data Availability

All sequencing and patient data can be made available upon reasonable request.
